# Association Between PM2.5 Air Pollution and All-Cause Mortality Among Older Mexican American Adults

**DOI:** 10.1093/geroni/igaf122.3281

**Published:** 2025-12-31

**Authors:** Sirena Gutierrez, Phillip Cantu, Kyriakos Markides, Sadaf Milani

**Affiliations:** University of California, San Francisco, San Francisco, California, United States; UTMB Health, The University of Texas Medical Branch, Galveston, Texas, United States; UTMB Health, The University of Texas Medical Branch, Galveston, Texas, United States; University of Texas Medical Branch, Galveston, Texas, United States

## Abstract

Exposure to air pollution, including fine particulate matter (PM2.5), is associated with higher all-cause mortality rates. Racial and ethnic minority groups, including Mexican American adults, are disproportionately exposed to higher levels of air pollution. However, it remains unclear whether this increased exposure translates into a higher mortality risk among older Mexican American adults. This analysis included participants ages 74+ from Wave 5 (2004-2005) of the Hispanic Established Population for the Epidemiological Study of Elderly. Baseline PM2.5 was measured as the annual average at the census tract level, and mortality was established through National Death Index linkages up to December 31, 2018. Cox proportional hazard models were used to estimate the hazard ratio for all-cause mortality, adjusting for sociodemographic characteristics, including age, gender, educational attainment, marital status, nativity, state of residence, and urbanicity. Our sample (n = 1,990) had a mean age of 81.9 years [standard deviation (SD): 5.1] and was 61.6% female. Participants lived in census tracts with average PM2.5 concentration of 10.4 μg/m3 (SD: 3.4, range: 5.1-20.0). We analyzed 13,370 person-years with 1,341 deaths. For every 5-μg/m3 increase in PM2.5 exposure there was a 5.4% increase in all-cause mortality, but the estimate was not statistically significant (95% confidence interval: -6.9% to 19.4%). Future work will include longitudinal exposure data and explore effect heterogeneity to understand the long-term pathways through which air pollution impacts mortality in older Mexican Americans.

